# Effectiveness of Diet Habits and Active Life in Vocational Training for Higher Technician in Dietetics: Contrast between the Traditional Method and the Digital Resources

**DOI:** 10.3390/nu12113475

**Published:** 2020-11-12

**Authors:** José-Antonio Marín-Marín, Rebeca Soler-Costa, Antonio-José Moreno-Guerrero, Jesús López-Belmonte

**Affiliations:** 1 Department of Didactics and School Organization, University of Granada, 18071 Granada, Spain; jmarin@ugr.es (J.-A.M.-M.); ajmoreno@ugr.es (A.-J.M.-G.); jesuslopez@ugr.es (J.L.-B.); 2 Department of Educational Sciences, University of Zaragoza, 50009 Zaragoza, Spain

**Keywords:** diet habits, nutrition, educational innovation, nutritional knowledge, healthy habits, active lifestyles, mobile apps, digital platforms, vocational training

## Abstract

Vocational training of students in diet habits and active lifestyle habits has recently become an important issue, given the health problems caused as a result of a poor diet. The objective of this study is to analyze the effectiveness of different training actions (traditional method and digital resources) carried out in a program of dietary habits and active lifestyle at the vocational training stage. A quasi-experimental design of the pre-post type was developed. A sample of 177 participants was chosen. The instrument to collect the data was the validated ECHAES questionnaire. The results show that all study groups demonstrated similar averages across all dimensions, except in the digital resource post-test design, where the averages were higher than the rest. There was a significant relationship between the traditional teaching method and the post-test digital resources design in all dimensions. There was also a significant relationship between the pre-test and post-test of the traditional teaching method and the digital resource group in the dimensions. It can be concluded that both the traditional and the innovative method lead to learning in the vocational training student, although the values achieved by the group where the innovative method was adopted were much higher than in the traditional group.

## 1. Introduction

Nutrition education programs can help to improve dietary and nutritional patterns in adolescents [1], generating a positive impact on the knowledge of young people about healthy eating [2]. In addition, they promote active lifestyles that prevent disease [3]. For this reason, pedagogical actions in which healthy diets are promoted and are also associated with moderate physical activity improve the quality of life [4], even having a positive impact on the academic performance of higher education students, obtaining better results than students with poor nutritional habits [5]. For this reason, it is necessary to offer students, at any educational stage, adequate nutritional knowledge [6,7], consolidating healthy lifestyles [8]. It is noteworthy that students’ knowledge about healthy habits is achieved thanks to self-learning, or thanks to the recommendations of people to whom they are close [9,10]. Therefore, quality dietary training given by qualified professionals can increase the indicators of reduction in obesity and can facilitate the improvement of psychological factors [11].

There are no studies in the impact literature that analyze the effectiveness of dietary education at the vocational training stage. On the other hand, this aspect is observed in other educational stages. Primary education is the most important stage for implementing training programs linked to the development of appropriate dietary patterns [12]. This is justified by the constant transformations which the body undergoes in the first years of life [13] and in the development of the students’ personality in primary education [14], in order to establish healthy diet habits that have a beneficial impact on their diet and that continue throughout life [15]. In this respect, it is important that teachers in educational establishments and staff in charge of school canteens receive specific training in the preparation of appropriate diets, in which there is control over energy-containing foods, fats in general and saturated fatty acids in particular [16].

Particularly, traditional programs to promote healthy eating habits have been found to be ineffective in improving people’s eating patterns. Therefore, alternatives for carrying out this training have been considered, incorporating electronic resources such as mobile devices, which have proven to be more effective in monitoring diets [17,18]. In this sense, innovative food training programs are being carried out through educational technology based on e-learning facilitating access for the implementation of nutritional training programs [19]. Moreover, thanks to advances in technology, it is possible to perform dietary interventions using mobile applications [20], as well as health education through emerging technologies such as augmented reality [21].

Recent studies in children and adolescents [22] and university students [23] on the incorporation of computer games to increase nutritional knowledge and support healthier food choices have provided results that show how students, of any educational stage, who interact with computer games develop more effective learning in the acquisition of nutritional knowledge and how this digital environment can support healthier food choices in young people. In this sense, there are training experiences in which the use of educational applications through mobile devices such as smartphones has been incorporated into the ordinary classroom, where students learn about healthy eating through blended learning that includes both classroom instruction and self-directed e-learning through applications that excite them [24]. The World Wide Web (WWW) itself is offered as a useful tool for interactive nutrition education, where it can lead to important changes in people’s behavior [25]. In this way, nutrition education is facilitated and enhanced by the benefits of technology, providing rich learning environments while maintaining a high level of individual satisfaction [26]. Technology, as well as the use of digital resources with an educational and pedagogical purpose, favors the learning process exponentially. All this affects different aspects such as motivation, the interactions produced between educational agents, the assimilation of the content as well as the final learning results [27,28].

In relation to the question of how education can contribute to alleviating this problem, the rate of responses found in recent educational literature is extensive. Obviously, the starting point is to promote good nutritional habits with specific training programs and courses [1,2]. The development of training actions has high benefits since it contributes to good nutrition and the acquisition of active lifestyles that counteract the appearance of different diseases and even yield improvements in the academic performance of students [3,4,5]. Hence, the emphasis is on this type of training in primary education, as it is an optimal stage for the students’ developmental level and can act as a measure of prevention and acquisition of important habits [14,15].

In particular, the vocational training stage is conceived under the principles of innovation with the purpose of disseminating knowledge from a new perspective; it is more attractive and linked to the new environment typical of a technological era [29,30]. Specifically, in nutrition education, the literature states that creativity and innovation are two fundamental aspects for teaching dietary habits and active lifestyles [31]. The realization of innovative practices in future educators and teachers is of great relevance for the promotion and transmission of the values and principles of innovation. All this is accompanied by the aim that future professionals who are in the classrooms of tomorrow carry out a new, innovative, attractive and effective teaching and learning process [12].

At present, as the number of obese people grows, increasingly in those of younger ages, the promotion of these healthy habits from an innovative perspective is a promising strategy to address these issues [32]. The use of technological resources to promote healthy habits in students and their families in order to combat sedentary lifestyles and improve eating patterns is highly advisable [15,16]. In fact, there are many innovation programs in food education and even dietary monitoring through e-learning and augmented reality and their benefits are considerable [17,18,20].

Diet education and active lifestyles have been revealed as topics of great interest by the scientific community given the characteristics of society and the continuous growth of sedentary lifestyles [33]. For this reason, this training has led researchers to carry out innovative studies considering their implementation in various groups [34,35]. This study aims to continue the path of innovation reflected in the literature at this educational stage. The Higher Technician in Dietetics was selected to verify the inclusion of digital resources in this vocational training degree. This is justified by the absence of studies that support its effectiveness in this very precise context. In addition, this research will contribute to the promotion of innovation in learning spaces. Specifically, it will expand the knowledge on the use of digital resources in the Higher Technician in Dietetics, since the literature does not contain many studies. The present research aims to analyze and compare the effectiveness of two teaching methods, one traditional and the other through digital resources, in a program for the development of dietary habits and the promotion of active lifestyles in students pursuing a Higher Technician career in Dietetics in the professional training stage. Likewise, it is intended to determine the scope of each training method in each of the dimensions studied. From these general statements, the following research questions (RQ) arise to conduct the study:

- Does the application of digital resources have more influence than a traditional method on food concern, on knowledge of dietary guidelines, on eating habits outside of school hours and on nutritional awareness of Higher Technician in Dietetics students?
- Does the application of digital resources have more influence than a traditional method on behaviors, eating attitudes and on the recommendations on physical activity of the Higher Technician in Dietetics students?
- Does the application of digital resources have more influence than a traditional method in the consumption of meat, dairy and cereal products in Higher Technician in Dietetics students?

The following hypotheses derive from these research questions:

-
  **Hypotheses** **1 (H1).** The use of digital resources to work on diet habits and promote active lifestyles is effective compared to a traditional teaching method in students of the Higher Technician in Dietetics.
-
  **Hypotheses** **2 (H2).** The use of digital resources to work on diet habits and promote active lifestyles is not effective compared to a traditional teaching method in students of the Higher Technician in Dietetics.

## 2. Materials and Methods

### 2.1. Research Design

The study has been developed using a quantitative methodology. The research design carried out has been quasi-experimental and of a pre-post, descriptive and correlational nature. The considerations of experts in this type of methodology have been taken into account for a correct investigative action [36,37]. In addition, the analysis structure of previously reported studies of impact databases has been followed to carry out a method validated by science [38,39,40].

**Figure 1:**
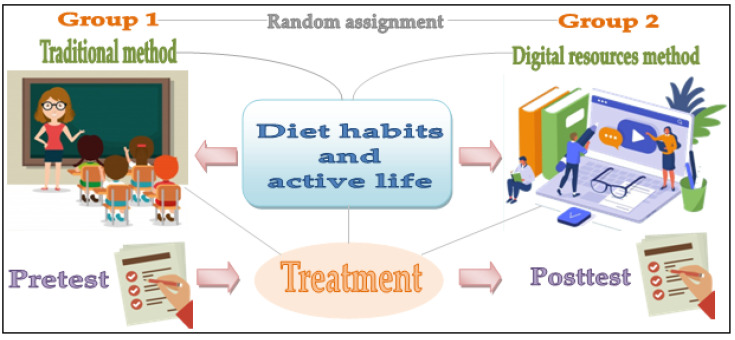
Graphic description of the research design.

In particular, in this research, a process of formative experimentation has been carried out—that is, through the application of two different instructive actions [41,42], such as the traditional method and the use of digital resources. This study has led to the configuration of two groups of a different nature, one being the traditional teaching method and the other being the digital resources method (Figure 1). Likewise, two main variables have been defined. The independent variable has been the type of training action carried out on the participants. The dependent variable has been the effect caused in the dimensions analyzed.

The research was carried out in 2019 in a vocational training educational center located in Southern Spain. The experimentation was carried out in the degree of Higher Technician in Dietetics. Before starting the research, the application for the ethics statement was made, being accepted. Access to the institution was not a problem due to the close relationship between the researchers and the teachers of this training center. Despite this, informed consent was obtained from both the educational center and the participants. All this was done with the purpose of carrying out the investigation in compliance with ethical considerations. Both the teaching staff and all study participants were aware of the research objectives at all times. Likewise, the students who participated in the study did so voluntarily.

Specifically, the experimentation took place at the training level. Training was developed centered on a work unit consisting of eight sessions on diet habits and active lifestyle using two different methodologies. The contents taught were nutritional needs and recommendations; balanced diet; public health. For this, two groups of students were taken and in each one of them a specific teaching action was applied. The traditional teaching method carried out an instructive process using a traditional methodology [36]. This type of training was characterized by not using any type of technological resource. The teacher was the main protagonist of the teaching process. The professor transmitted the contents in an expository and unilateral way. The student was limited to paying attention to the teacher’s explanations and performing the tasks. Instead, the digital resources group carried out a training plan through the use of digital resources. In this case, an ad hoc mobile application designed by the computer science department was used based on the requirements of the unit taught. In addition, an LMS (learning management system) platform was used where the contents were hosted in a complementary way so that the student could consult the information [42]. In this training modality, students became the protagonists of the instructional process. The teacher simply limited himself to guiding the students and solving the doubts caused in the interaction with the mobile application or with the access or understanding of the digital contents of the platform. Students could use their own resources (tablets, smartphones) through a BYOD (bring-your-own-device) program [41]. In addition, the students accepted the conditions for critical and responsible use of the technology employed. The educational center offered resources for those students who did not have the necessary technological material. The assignment of the group of students to the type of training occurred randomly.

In the different groups, the work unit was taught by the same teacher in order to avoid any alteration or bias produced by the intervention of different professionals. Once the training was completed, the students filled out a questionnaire and data analysis began.

### 2.2. Participants

A total of 177 vocational training students who were studying on the Higher Technician in Dietetics course participated in the study. The rationale for selecting this population to carry out the study focused on several reasons. The first reason focused on the knowledge about the type of teaching methodology most appropriate for the degree in question that was analyzed. The second reason focused on the awareness of students about the possibility of applying innovative methodologies for the work of didactic contents. This last reason was fundamental because, in the future, they will be educators and transmitters of knowledge to new generations of students. The sampling technique was intentional given the ease of access to the participants. In this case, the number of subjects did not influence the type of study as revealed by the experts [43,44], the sample size of the present study being adequate.

At the social level, the characteristics of the sample were as follows: 64.4% were men and the rest women, with a mean age of 19 years (SD = 1.13). The students were divided into two study groups. One of the groups, in which the teaching method based on the traditional teaching method (TT) was developed, consisted of 89 students. In the other group, the teaching method based on digital resources (DR) was developed and consisted of 88 students. In both pre-test and post-test, design measures were applied with the same instrument, which is specified below.

### 2.3. Instrument

Specifically, to collect the data of the participants, the validated questionnaire ECHAES [45] was used as an instrument. This questionnaire collected information about the attitudes and behaviors of the participants regarding their dietary habits and lifestyle. This tool was made up of 35 questions configured on a 5-point Likert scale. The different questions were classified into 10 dimensions (1—food concern = five items; 2—food guidelines = six items; 3—eating out of hours = six items; 4—food awareness = three items; 5—eating behaviors—sedentary = five items; 6—meat products = two items; 7—dairy and cereals = two items; 8—recommendations for physical activity = three items; 9—eating attitude = two items; 10—sedentary activity = one item). According to its authors, the validation was produced by means of a factor analysis using the principal components method with varimax rotation. The Kaiser–Meyer–Olkin (KMO) test reached a score of 0.802 and the Bartlett sphericity test was relevant (X^2^ = 3181.055; df = 595; *p* < 0.0001). Likewise, the instrument obtained adequate reliability, revealed by Cronbach’s alpha statistic (α = 0.815). Specifically, in the context in which this questionnaire was applied, the values achieved in each of the study dimensions, both in KMO and α, were food concerns (KMO = 0.829; α = 0.843); food guide (KMO = 0.813; α = 0.826); out-of-hours-feed (KMO = 0.801; α = 0.807); food awareness (KMO = 0.789; α = 0.799); sedentary eating (KMO = 0.806; α = 0.815); meat producer (KMO = 0.811; α = 0.817); dairy and cereals (KMO = 0.807; α = 0.812); physical activity (KMO = 0.823; α = 0.832); eating attitude (KMO = 0.794; α = 0.801); sedentary activity (KMO = 0.751; α = 0.802).

In relation to each of the dimensions, it can be indicated that each of them refers to:

- Food concern: measures students’ attitudes towards and interest in a healthy weight and healthy food consumption.
- Food guide: identifies people’s knowledge of the dietary guidelines of the Ministry of Health, about the consumption of foods such as fruits, vegetables, fish and legumes.
- Out of hours feed: measures food consumption between meals, which is associated with psychological behaviors.
- Food awareness: measures the subject’s ability to control food intake in order to have adequate nutrition in terms of quantity.
- Sedentary eating: measures the daily inactivity of students and its relationship with the consumption of poorly nutritious and balanced foods.
- Meat products: measures attitudes and behaviors towards the intake of protein products.
- Dairy and cereals: measures students’ attitudes and behaviors towards the consumption of dairy and cereals.
- Physical activity: measures the attitudes and behaviors of students to avoid sedentary lifestyle. It also brings together water consumption.
- Eating attitude: measures the physical and temporal attitude of the students toward the act of eating.
- Sedentary activity: evaluates the time for which the student is in a state of rest or inactivity.

### 2.4. Data Analysis

In order to carry out an in-depth analysis that allowed the achievement of the objectives and answered the questions asked, the Statistical Package for the Social Sciences (SPSS) v25 (IBM Corp., Armonk, NY, USA) program was used. Statistics such as the mean (M), standard deviation (SD) and the standard error of the mean (SE) were included in the analysis. Skewness (S_kw_) and kurtosis (K_me_) tests were used to reveal the trend of the distribution. In addition, the coefficient of variation (CV) was analyzed to identify response dispersion. These statistics were applied to identify whether or not it was feasible to use the *t*-Student test. The *t*-Student test (*t_n_*_1+*n*2−2_) was used to compare the means between the groups. Cohen’s d and biserial correlation (*r_xy_*) were used to determine the size of the effect achieved after the training process. The analysis was developed taking into account values of *p* < 0.05 as statistically significant differences.

All the values of asymmetry and kurtosis were in the range of ±1.96, being thus the premises marked by [42] for a normal distribution.

The degree of independence of the results obtained was measured with the Student T statistic, although from two different perspectives. Independent samples were analyzed—that is, a comparison was made between the traditional teaching method and the digital resources groups, both in the pre-test and the post-test.

## 3. Results

The comparison of means of the different study dimensions shows an entirely different response trend in the digital resources post-test design with respect to the rest of the group. Moreover, within each of the groups, one dimension is not observed to be more valued than another. What is observed is that in each of the established groups, the response trend is similar in the dimensions of the study. Response dispersion has been calculated using the coefficient of variation. According to the results obtained, this index is low in most dimensions, although there are some that are in a medium range [46]. Both in the traditional pre-test design and in the digital resources post-test design, the response trend is mainly placid. On the other hand, in the other groups, both in the traditional post-test and in the digital resources pre-test, the response tendency is leptocurtic. Exceptionally, a mesocuric type of kurtosis is shown. The comparison of means of the different study dimensions shows an entirely different response trend in the digital resources post-test design with respect to the rest of the group. Moreover, within each of the groups, one dimension is not observed to be more valued than another. What is observed is that in each of the established groups, the response trend is similar in the dimensions of the study (Table 1).

There are slight differences in means between pre-test and post-test measures in the TT group, in favor of post-test measures. In contrast, between the pre-test and post-test measures in the DR group, there are wider differences in the post-test measures.

The independence of the two samples is such according to the random assignment to the treatments, so the test confirms this situation in the pre-test phase, while the non-independence on the post-test in this case signals a situation of a common background generated by the two different training methods, thus confirming the effectiveness of both. This is the case for the dimensions food concerns, food guide, out-of-hours-feed, food awareness, sedentary eating, meat products, dairy and cereals, physical activity, eating attitudes and sedentary activity of the measures adopted in the pre-test in both designs. Thus, the biserial correlation indicates that there is a mean strength of association in all the study dimensions in the post-test tests of the traditional teaching method and the digital resources group. The effect size is very low in all study dimensions where significant relationships have been observed (Table 2).

On the other hand, related samples have been analyzed, i.e., between the pre-test and post-test of the traditional teaching method and digital resources groups. In this case, it is observed that there is a relationship of significance in all the study dimensions in both groups (Table 3). Hence, the non-independence between pre-test and post-test demonstrates that, although both methods are effective, the strength of effectiveness is different and it is in favor of the DR vs. the TT. This indicates that the methodological procedures developed in each of the groups have been effective in generating learning in the students. Thus, the statistical data given by the value of t in one group and another group vary considerably in each of the dimensions. Obviously, this shows greater influence in the digital resources group than in the traditional teaching method.

## 4. Discussion

This study has analyzed the influence that two pedagogical methods—in this case, traditional teaching and teaching through the use of technological resources—have on the acquisition of content related to diet habits and active lifestyles. This content has been developed in vocational training, specifically in the Higher Level Training Cycle of Dietetics, in the Spanish context. After analyzing the results obtained, it can be indicated that the pedagogical method based on the use of technological resources generates better learning and better acquisition of the content, as was obtained in recent studies [47,48]. That is, the application of an active and innovative teaching method leads to substantial improvements in the learning of students of vocational training in the Higher Degree of Dietetics. This coincides with other studies, where the application of active teaching methods led to improvements in student learning [29,30].

Overall, the results of the present study support the validity of Hypothesis 1 for all three research questions, thus demonstrating that the pedagogical method based on digital resources is much more effective than the traditional method. In fact, the following assertions can be made:

(a) Analyzing in more detail the dimensions of studies related to diet habits and active lifestyles before the learning course, these being food concerns, food guide, out-of-hour-feed, food awareness, sedentary eating, meat products, dairy and cereal, physical activity, eating attitude and sedentary activity, we have observed similar results to other studies [31].
(b) Comparing pre-test and post-test tests within each of the approaches, an improvement in content acquisition was found, as revealed in other research [49]. However, the results were much higher in the group that received a teaching method based on digital resources than in the group that received a traditional teaching method. This element is evident in other research where the contrast between teaching methods is analyzed [39,40,41].
(c) When analyzing the results from the post-test, the method based on digital resources is effective for the acquisition of the content [50,51]. These results coincide with those presented in other studies, where two different pedagogical methods are applied, one traditional and the other innovative, obtaining better results for students where the innovative method has been used [29,30,31].

Consequently, the main result achieved in this research is to determine that the pedagogical method based on digital resources is much more effective than the traditional method in the education of vocational training students (educational stage in the Spanish educational system) in content related to diet habits and active lifestyles.

Therefore, it can be indicated that the research hypothesis raised in this study is fulfilled, which is that the use of digital resources to educate students about diet habits and active lifestyles is effective in comparison with a traditional teaching method in the students of the Higher Technician in Dietetics, given that it allows better academic values to be achieved in all the dimensions studied.

The main limitation of this study lies in the difficulty of comparing the findings obtained in this research with other related studies on the state of the art—specifically, the difficulty of discussing the results with impact studies in which the same population of vocational training students has been analyzed. Therefore, the scientific literature evidences a lack of studies similar to the present work. The results achieved and presented in this manuscript reveal unpublished data in the educational stage of vocational training. Therefore, this study acquires an exploratory component. This work will serve to start the path towards the study of dietary habits and active lifestyles in vocational training students. The main novelty and contribution of this research to the scientific literature focuses on the selected study population. The studies that involve students of Higher Technician of Vocational Training linked to formative methodological contrasts are scarce. Therefore, this work assumes a novel, innovative and exploratory nuance that represents an advance in science. Likewise, the results presented will allow for replications by other researchers interested in this field of knowledge.

With regard to implications, essentially, this research leads to practical implications of interest for various groups related to the educational field. This ranges from teachers, researchers and students to entities and institutions in charge of nutrition and the development of innovative educational tools. Among the findings more focused on teaching practice, the scientific literature shows the main pedagogical methods carried out in different contexts to promote healthy habits. Thus, we can observe the potential of educational technology in its application in learning processes, which is an issue that is currently attracting attention from different sectors, such as the World Health Organization.

## 5. Conclusions

In short, it should be noted that both the traditional and the innovative method generate learning in the student, although the values achieved by the group where the innovative method has been developed are much higher than in the traditional group. This indicates that the use of an innovative method for the teaching of diet habits and active lifestyles implies better acquisition and learning in students, especially when compared to traditional teaching methods.

As for future lines of research, it should be stated that it would be convenient to promote new e-learning programs that students can also use at home to ensure the learning and practice of healthy habits and offer them a tracking system to have the necessary feedback to redirect when possible. It would be convenient to train students in this issue to help them be active learners to apply this knowledge in their lifelong learning processes as responsible citizens.

Finally, the implementation of teaching and learning methods based on digital resources can promote the acquisition of content in relation to dietary education. This pedagogical method can serve to promote training actions in other teachers, and with it the improvement of teaching and learning processes typical of the technological era. Finally, the pedagogical action developed in this study can serve as a guide for other educational institutions where higher level vocational training is taught.

## Figures and Tables

**Table 1:** Results obtained for the dimensions of study in TT and DR group of vocational training.

Parameters											
Dimensions		Pre-Test					Post-Test				
		M	SD	S_kw_	K_me_	CV	M	SD	S_kw_	K_me_	CV
Food concerns	TT	2.37	0.958	0.458	−0.024	0.404	2.51	0.990	0.631	0.192	0.391
	DR	2.33	0.906	0.335	−0.158	0.388	3.39	0.988	−0.262	−0.244	0.291
Food guide	TT	2.39	0.973	0.649	0.278	0.407	2.54	0.966	0.815	0.596	0.380
	DR	2.35	0.858	0.250	−0.496	0.365	3.33	−0.956	−0.225	−0.120	0.287
Out-of-hours-feed	TT	2.35	0.966	0.790	0.832	0.411	2.48	0.967	0.898	0.798	0.389
	DR	2.31	0.835	0.213	−0.453	0.361	3.42	0.919	0.058	−0.792	0.268
Food awareness	TT	2.39	0.912	0.324	−0.232	0.381	2.47	0.955	0.523	0.215	0.386
	DR	2.36	0.973	0.663	0.351	0.412	3.41	0.942	−0.068	−0.548	0.276
Sedentary eating	TT	2.37	0.993	0.474	−0.221	0.418	2.61	0.973	0.715	0.304	0.372
	DR	2.28	0.909	0.528	0.452	0.398	3.43	0.944	−0.302	0.136	0.275
Meat products	TT	2.40	0.997	0.376	−0.320	0.415	2.54	0.978	0.596	0.201	0.385
	DR	2.39	0.988	0.617	0.180	0.413	3.47	0.883	−0.048	−0.202	0.254
Dairy and cereals	TT	2.43	0.824	−0.073	−0.531	0.339	2.53	0.955	0.359	0.107	0.377
	DR	2.27	0.919	0.426	0.281	0.404	3.44	0.945	−0.251	−0.206	0.274
Physical activity	TT	2.39	0.912	0.324	−0.232	0.381	2.46	0.954	0.396	−0.133	0.387
	DR	2.30	0.886	0.087	−0.761	0.385	3.55	0.896	0.057	−0.735	0.252
Eating attitude	TT	2.35	0.990	0.685	0.563	0.421	2.53	0.978	0.851	0.836	0.386
	DR	2.38	0.986	0.505	0.149	0.414	3.52	0.884	0.083	−0.687	0.251
Sedentary activity	TT	2.44	0.988	0.465	0.024	0.404	2.56	0.988	0.620	0.353	0.385
	DR	2.36	0.899	0.372	0.342	0.380	3.48	0.897	0.070	−0.713	0.257

Note. TT: traditional teaching method; DR: digital resources; M: mean; SD: standard deviation; S_kw_: skewness; K_me_: kurtosis; CV: coefficients of variation.

**Table 2:** Independent test for the two samples in the pre-test and post-test phase to highlight belonging to the same population determined by a common background.

	Dimensions	µ (TT-DR)	*t_n_*_1+*n*2−2_	df	*d*	*r_xy_*
Pret-test	Food concerns	0.041 (2.37–2.33)	n.s.	-	-	-
	Food guide	0.041 (2.39–2.35)	n.s.	-	-	-
	Out-of-hours-feed	0.041 (2.35–2.31)	n.s.	-	-	-
	Food awareness	0.030 (2.39–2.36)	n.s.	-	-	-
	Sedentary eating	0.087 (2.37–2.28)	n.s.	-	-	-
	Meat products	0.018 (2.40–2.39)	n.s.	-	-	-
	Dairy and cereals	0.154 (2.43–2.27)	n.s.	-	-	-
	Physical activity	0.098 (2.39–2.30)	n.s.	-	-	-
	Eating attitude	−0.027 (2.35–2.38)	n.s.	-	-	-
	Sedentary activity	0.075 (2.44–2.36)	n.s.	-	-	-
Post-test	Food concerns	−0.881 (2.51–3.39)	−5.925 **	175	−0.065	0.409
	Food guide	−0.790 (2.54–3.33)	−5.471 **	175	−0.086	0.382
	Out-of-hours-feed	−0.937 (2.48–3.42)	−6.611 **	175	−0.069	0.447
	Food awareness	−0.937 (2.47–3.41)	−6.572 **	175	−0.041	0.445
	Sedentary eating	−0.825 (2.61–3.43)	−5.725 **	175	−0.094	0.397
	Meat products	−0.927 (2.54–3.47)	−6.613 **	175	−0.067	0.447
	Dairy and cereals	−0.915 (2.53–3.44)	−6.408 **	175	−0.036	0.436
	Physical activity	−1.08 (2.46–3.55)	−7.796 **	175	−0.043	0.508
	Eating attitude	−0.995 (2.53–3.52)	−7.096 **	175	−0.070	0.473
	Sedentary activity	−0.915 (2.56–3.48)	−6.453 **	175	−0.047	0.438

Note. µ: difference in means between groups; *t_n_*_1+*n*2−2_: Student’s *t* test; df: degrees of freedom; *d*: Cohen’s d test; *r_xy_*: biserial correlation. ** correlation is significant at the 0.01 level, n.s. correlation not significant.

**Table 3:** Level of dependency between pre-test and post-test within TT and DR.

	Dimensions	µ (TT-DR)	*t_n_*_1+*n*2−2_	df	SD	SE
**Traditional teaching group**	Food concerns	−0.135 (2.37–2.51)	−3.703 **	88	0.343	0.036
	Food guide	−0.146 (2.39–2.54)	−3.880 **	88	0.355	0.038
	Out-of-hours-feed	−0.146 (2.39–2.54)	−3.880 **	88	0.355	0.038
	Food awareness	−0.079 (2.39–2.47)	−2.741 *	88	0.271	0.029
	Sedentary eating	−0.236 (2.37–2.61)	−5.213 **	88	0.427	0.045
	Meat products	−0.135 (2.40–2.54)	−3.703 **	88	0.343	0.036
	Dairy and cereals	−0.101 (2.43–2.53)	−3.146 *	88	0.303	0.032
	Physical activity	−0.067 (2.39–2.46)	−2.522 *	88	0.252	0.027
	Eating attitude	−0.180 (2.35–2.53)	−4.392 **	88	0.386	0.041
	Sedentary activity	−0.124 (2.44–2.56)	−3.523 **	88	0.331	0.035
**Digital resources group**	Food concerns	−1.05 (2.33–3.39)	−25.931 **	87	0.382	0.041
	Food guide	−0.977 (2.35–3.33)	−30.319 **	87	0.302	0.032
	Out-of-hours-feed	−1.11 (2.31–3.42)	−29.564 **	87	0.353	0.038
	Food awareness	−1.04 (2.36–3.41)	−26.609 **	87	0.369	0.039
	Sedentary eating	−1.14 (2.28–3.43)	−21.873 **	87	0.492	0.052
	Meat products	−1.08 (2.39–3.47)	−24.855 **	87	0.407	0.043
	Dairy and cereals	−1.17 (2.27–3.44)	−22.651 **	87	0.485	0.052
	Physical activity	−1.25 (2.30–3.55)	−26.926 **	87	0.435	0.046
	Eating attitude	−1.14 (2.38–3.52)	−24.300 **	87	0.443	0.047
	Sedentary activity	−1.11 (2.36–3.48)	−27.168 **	87	0.385	0.041

Note. μ: difference of means between groups; *t_n_*_1+*n*2−2_: Student’s *t* test; df: degrees of freedom; * correlation is significant at the 0.05 level, ** correlation is significant at the 0.01 level.
